# Supplementary material for: PseudoChecker2 and PseudoViz: automation and visualization of gene loss in the Genome Era
Source: Bioinform Adv. 2025 Nov 22;5(1):vbaf202. doi: 10.1093/bioadv/vbaf202 (PMC12679834; doi:10.1093/bioadv/vbaf202)
Supplement: vbaf202_Supplementary_Data [file vbaf202_supplementary_data.docx]

PseudoChecker2 and PseudoViz: automation and visualization of gene loss in the Genome Era

Rui Resende-Pinto^1,2^*, Raquel Ruivo^1^, Josefin Stiller^3^, Rute Fonseca^4^, L. Filipe C. Castro^1,2^*

^1^CIMAR/CIIMAR - Interdisciplinary Centre of Marine and Environmental Research, University of Porto, Avenida General Norton de Matos, S/N, 4450-208 Matosinhos, Portugal.

^2^FCUP - Department of Biology, Faculty of Sciences, University of Porto (U. Porto), Rua do Campo Alegre, Porto, Portugal.

^3^Department of Biology, University of Copenhagen, Universitetsparken 15, 2100 Copenhagen, Denmark

^4^Center for Global Mountain Biodiversity, GLOBE Institute, University of Copenhagen, Universitetsparken 15, 2100 Copenhagen, Denmark

*Correspondence to: rui.pinto@ciimar.up.pt and filipe.castro@ciimar.up.pt, +351 223401800, ORCID ID: 0000-0001-7697-386X

**PseudoViz interface and usage**


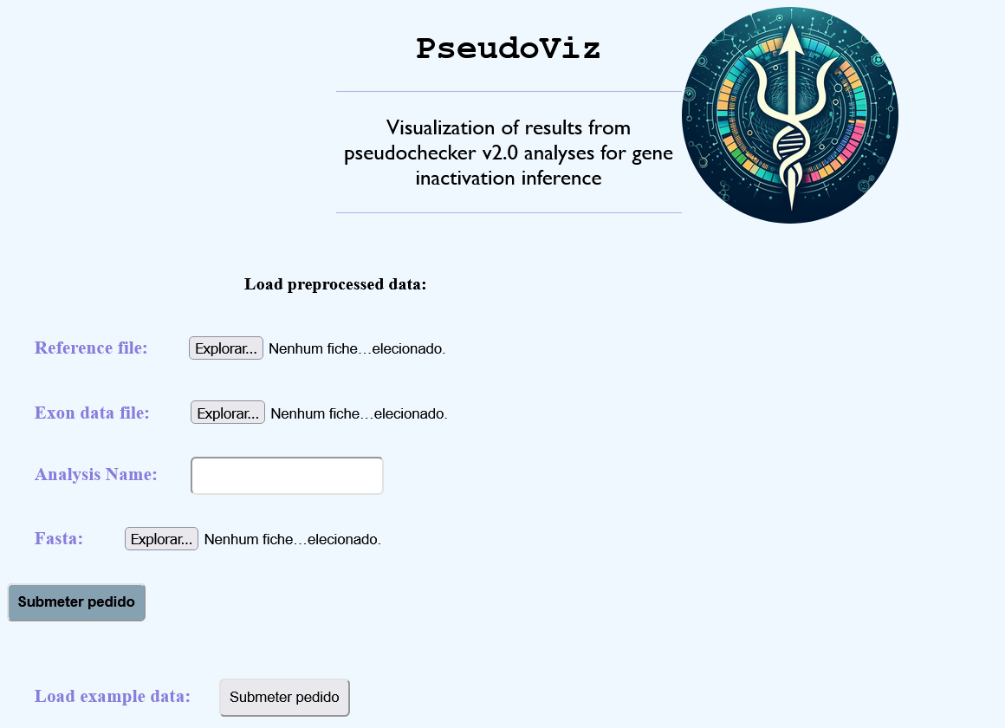


**SFig. 1. Data input** Homepage of PseudoViz in which the user can submit the output of their PseudoChecker2 analysis or check the CYP2J19 example data. PseudoViz initially requires minimal user input: the json file with information on the resulting exon alignments from PseudoChecker2’s main analysis (exon_alns.json), the reference fasta, with exons and CDS, used for PseudoChecker2’s main analysis, an adequate name for the analysis and the fasta file with the genomic target sequences used for PseudoChecker2’s main analysis (optional, only needed for the exon painter view)

*SFig. SEQ Figure \* ARABIC 1- The landing page of PseudoViz, in which the user provides the necessary input.*


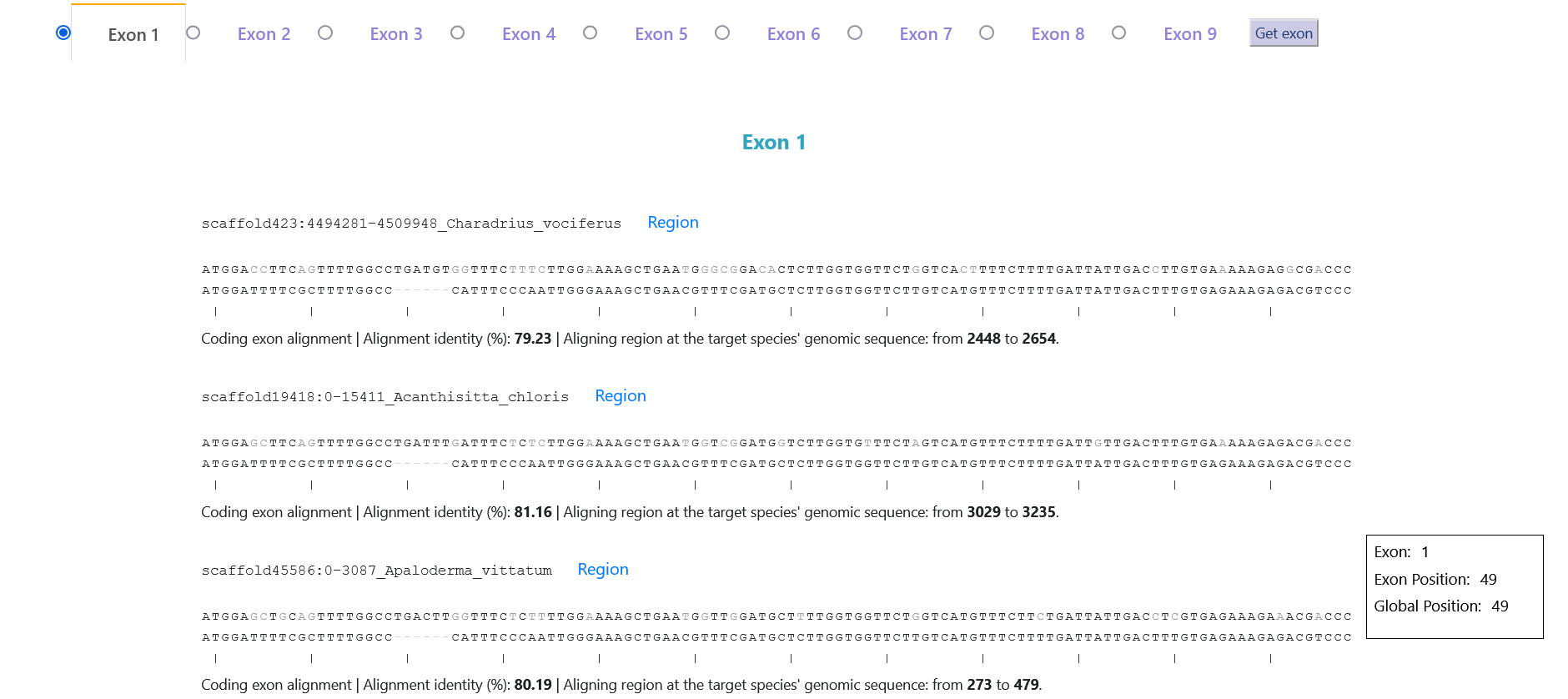


**SFig. 2. Exon alignments page** The PseudoViz page that displays alignments of orthologous exons in different species to a single exon of the reference ortholog. Here are shown sequences pertaining to the CYP2J19 dataset. By clicking on the Region link on each of the target displays in this page, the user is redirected to a page showing the position of exons in genomic region (Exon Painter page), where the exon sequences are highlighted in the middle of the target sequences. Splice sites are also highlighted in green.


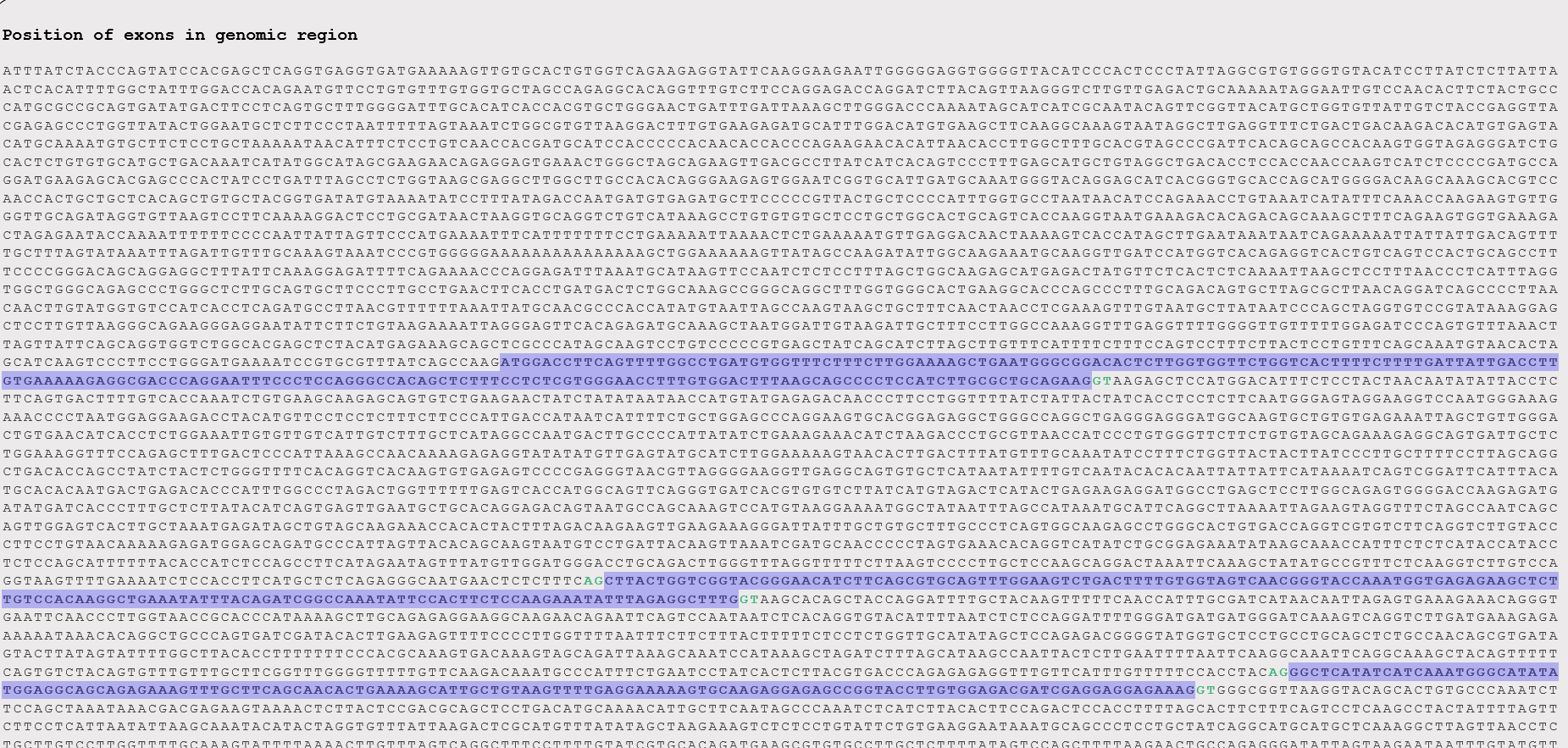


**SFig. 3. Exon painter page** displaying the orthologous exons within one of the genomic regions submitted to PseudoChecker2 analysis from the CYP2J19 dataset.

**SFig. 4. MACSE MSA page** MACSE alignment in PseudoViz, displaying aligned sequences of the CYP2J19 dataset. In the macse alignments page, the user can submit the results of multiple sequence alignments done with PseudoChecker2.0. The user can display at the same time more than one MSA. This view intends to maintain a similar display to that of the first PseudoChecker.


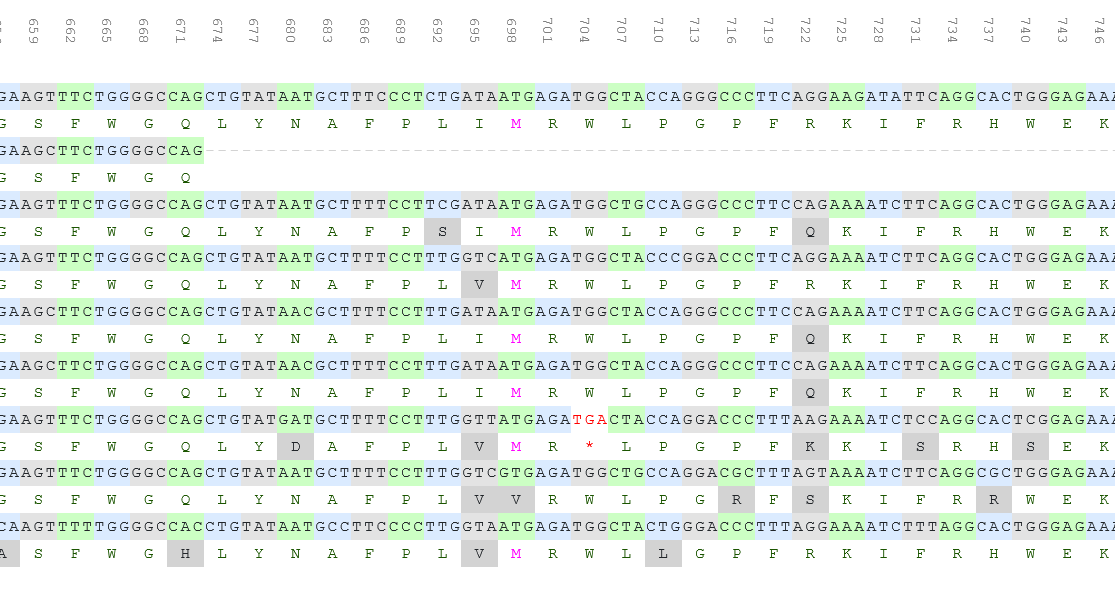

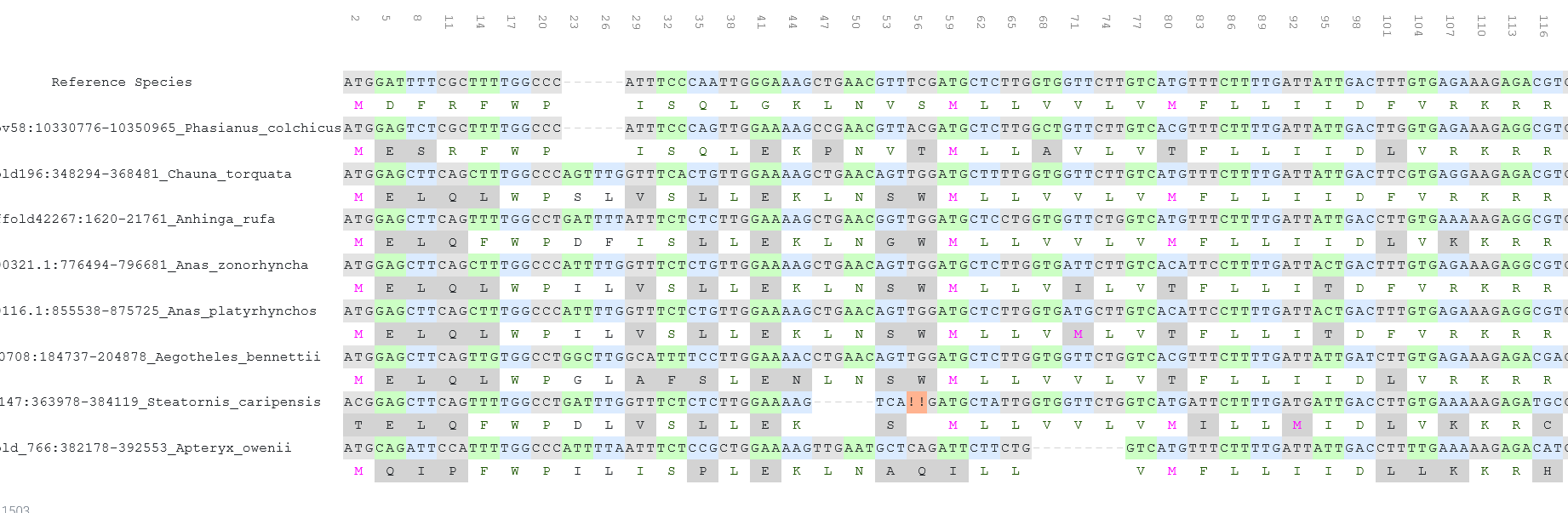


*SFig. SEQ Figure \* ARABIC 5 - MACSE alignment of several predicted orthologous sequences of CYP2J19. The Reference Species is G. gallus. It is possible to see an insertion of 2bp in S. caripensis in position 55 of the alignment (exon 1) and a premature stop codon in A. bennettii in position 703 of the alignment (exon 5).*


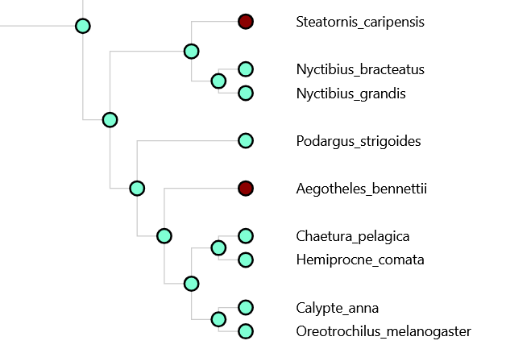

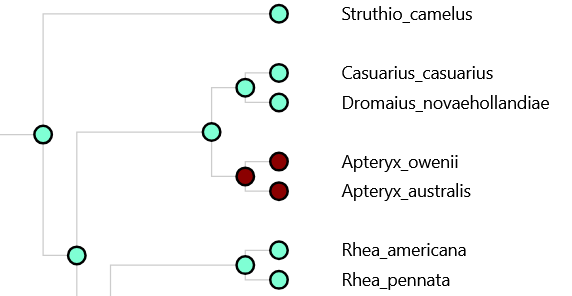

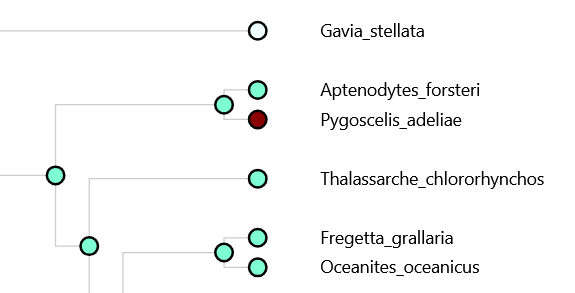

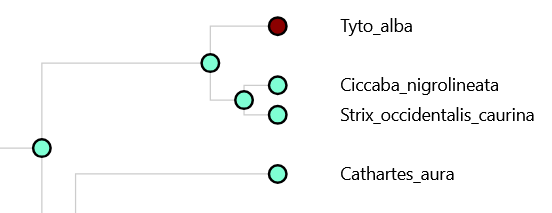


**SFig. 5. Phylogeny-based visualization** Dendrograms displaying the calculated PseudoIndex, including PseudoIndex 5 (maximum score) for the CYP2J19 ortholog in a) Pygoscelis adeliae, b) Tyto alba, c) Apteryx genus, d) S. caripensis and A. bennettii. In the dendrogram page, the user can submit a json file created from the PseudoChecker2 output folder using the pseudochecker_phylogeny.py and the page will display a dendrogram displaying a provided phylogeny (branch lengths are not accounted for) in which the nodes’ color corresponds to the assigned PseudoIndex metric. The PseudoIndex of the leaf nodes corresponds to the PseudoIndex of the target sequence to which that node corresponds. The PseudoIndex of the middle nodes corresponds to the minimum PseudoIndex of the child nodes. This is designed to provide an easy overview of the erosion of the gene along a given phylogeny. When hovering over the nodes, a small text box will display the present mutations in the target or, in the case of the middle nodes, the mutations that are present in all its child nodes.

**Application: inactivation of the *CYP2J19* gene in birds**

To test PseudoChecker2 and PseudoViz, we assessed the coding status of CYP2J19, a gene that is responsible for the production of red retinal oil droplets in birds and turtles (Lopes et al., 2016; Mundy et al., 2016; Twyman et al., 2016) and has been described as lost in penguins, owls and kiwis (Emerling, 2018). These bird lineages display adaptations to dim light and negligible levels of red oil droplets (Bowmaker & Martin, 1978, 1985; Gondo & Ando, 1995; Yew et al., 1977). We used a previously published dataset, consisting of 363 genomes from 92.4% of bird families from the Bird 10,000 Genomes (B10K) Project (Feng et al., 2020). This case study, where hundreds of genomes need to be analysed, illustrates the advantage of the command-line-based tool presented here over the web-tool, where each species/genome would have to be manually annotated. Annotations of protein-coding genes provided by the B10K consortium were used to extract the genomic regions expected to have the ortholog of CYP2J19 and to create the reference using AGAT v1.0.0. In cases where the ortholog was annotated, coordinates starting from 2000 base pairs (bp) upstream of the start of the annotated gene to 2000 bp downstream from the end were extracted using samtools v1.15.1 faidx (Li et al., 2009). When the ortholog was not annotated but a contiguous syntenic region could be obtained via the annotated orthologs of the flanking genes (the genes immediately downstream and upstream of the target in the genomic reference), the genomic region was extracted, with coordinates from the end of the first upstream ortholog to the start of the first downstream ortholog. Additionally, we used blastn v2.12.0 (Altschul et al., 1990) to search for a CYP2J19 ortholog in the Caprimulgus europaeus genome (GCA_907165065.1), which was then included in the downstream analyses with the rest of the dataset.

Using PseudoChecker2, using G. gallus (XM_422553.6) as the reference, we were able to retrieve inactivating mutations in the same lineages as Emerling (2018), namely in penguins, owls and kiwis. Of note, we also identified previously unreported cases of gene inactivating mutations in the nightjar A. bennettii (a premature stop codon in exon 5 at position 697 of the coding sequence) and the oilbird S. caripensis (a deletion of 2bp at position 26 of the first exon; Strisores; SFig. 7). This last mutation is just before a possible start codon in the first exon. We did not find inactivating mutations in any of the other Strisores of the B10K dataset or Caprimulgus europaeus.

**
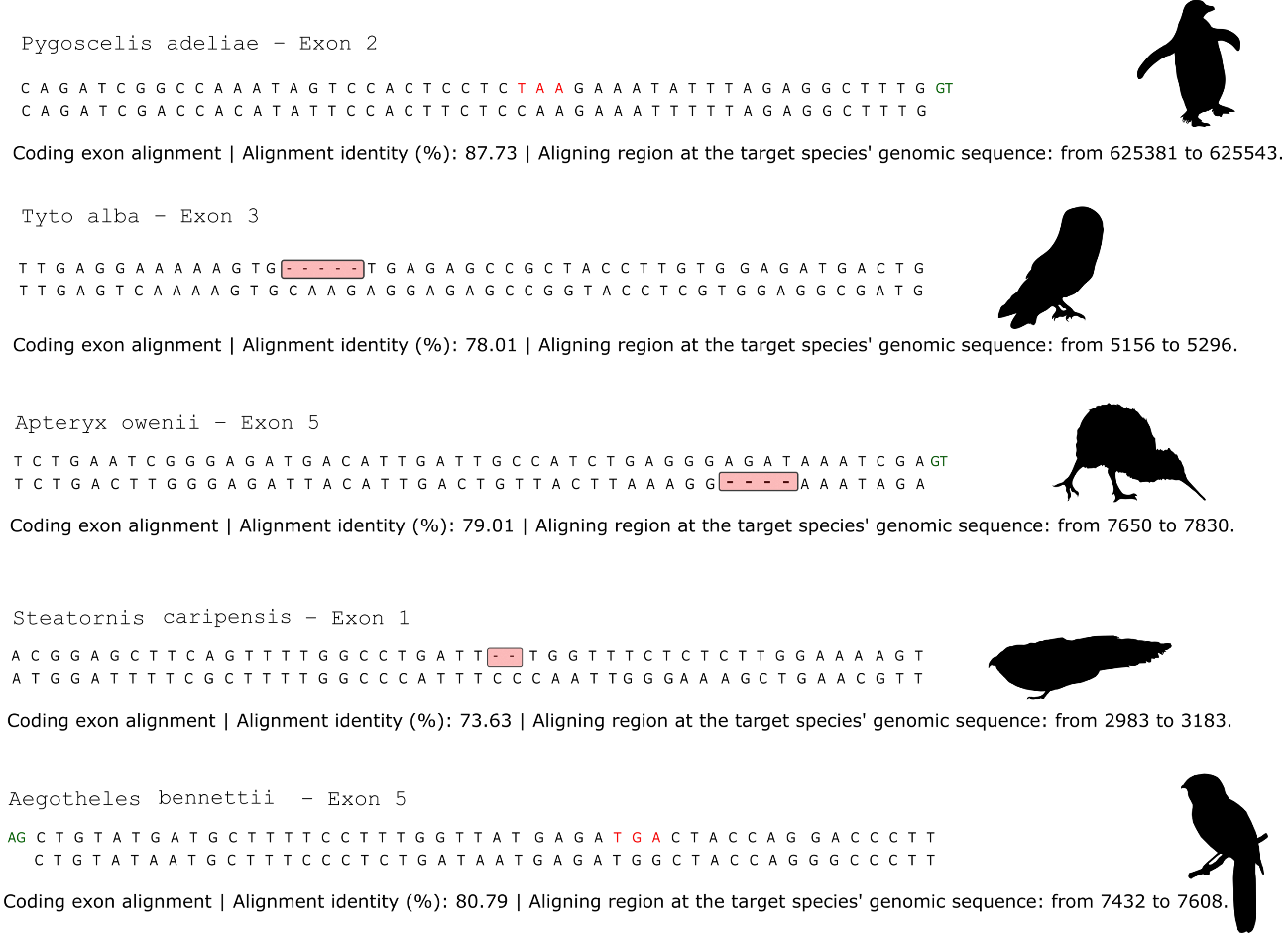
**

**SFig. 7** - Adapted exon alignment display from PseudoViz showing inactivating mutations in the gene CYP2J19 across birds, specifically in a penguin (premature stop codon in exon 2 at position 349 of the reference coding sequence; Pygoscelis adeliae, Sphenisciformes), an owl (5bp deletion at position 96 of exon 3; Tyto alba, Strigiformes), a kiwi (4bp deletion at position 167 of exon 5; Apteryx owenii, Apterygiformes), the oilbird (2bp deletion at position 26 of exon 1; Steatornis caripensis, Strisores) and a nightjar (premature stop codon at position 697 of the reference coding sequence; Aegotheles bennettii, Strisores). The gene is inactivated in four orders of birds and possibly twice independently within Strisores. G. gallus was used as the reference sequence and the respective exonic sequences are displayed on the bottom of each alignment.

**References**

Altschul,S. F. et al. (1990). Basic local alignment search tool. Journal of Molecular Biology, 215(3), 403–410. https://doi.org/10.1016/S0022-2836(05)80360-2

Alves,L. Q. et al. (2020). PseudoChecker: An integrated online platform for gene inactivation inference. Nucleic Acids Research, 48(W1), W321–W331. https://doi.org/10.1093/nar/gkaa408

Chen,A et al. (2019). Total-Evidence Framework Reveals Complex Morphological Evolution in Nightbirds (Strisores). Diversity, 11(9), Artigo 9. https://doi.org/10.3390/d11090143

Cock, P. J. A. et al. (2009). Biopython: Freely available Python tools for computational molecular biology and bioinformatics. Bioinformatics, 25(11), 1422–1423. <https://doi.org/10.1093/bioinformatics/btp163>

Dainat,J. et al. (2023) AGAT: Another Gff Analysis Toolkit to handle annotations in any GTF/GFF format, https://doi.org/10.5281/zenodo.4205393

Needleman, S. B., & Wunsch, C. D. (1970). A general method applicable to the search for similarities in the amino acid sequence of two proteins. Journal of Molecular Biology, 48(3), 443–453. https://doi.org/10.1016/0022-2836(70)90057-4

Stiller, J. et al. (2024). Complexity of avian evolution revealed by family-level genomes. Nature, 629(8013), 851–860. https://doi.org/10.1038/s41586-024-07323-1
